# NGS-defined measurable residual disease (MRD) after initial chemotherapy as a prognostic biomarker for acute myeloid leukemia

**DOI:** 10.1038/s41408-023-00833-7

**Published:** 2023-04-24

**Authors:** Yonghong Li, Jose Solis-Ruiz, Fei Yang, Nicola Long, Carmen H. Tong, Felicitas L. Lacbawan, Frederick K. Racke, Richard D. Press

**Affiliations:** 1 Quest Diagnostics, San Juan Capistrano, CA USA; 2 Department of Pathology, Oregon Health & Science University, Portland, OR USA; 3 Knight Cancer Institute, Oregon Health & Science University, Portland, OR USA

**Keywords:** Acute myeloid leukaemia, Risk factors

## Abstract

Treated AML patients often have measurable residual disease (MRD) due to persisting low-level clones. This study assessed whether residual post-treatment somatic mutations, detected by NGS, were significantly prognostic for subsequent clinical outcomes. AML patients (*n* = 128) underwent both pre-and post-treatment testing with the same 42-gene MRD-validated NGS assay. After induction, 59 (46%) patients were mutation-negative (0.0024 VAF detection limit) and 69 (54%) had ≥1 persisting NGS-detectable mutation. Compared with NGS-negative patients, NGS-positive patients had shorter overall survival (17 months versus median not reached; *P* = 0.004; hazard ratio = 2.2 [95% CI: 1.3–3.7]) and a shorter time to relapse (14 months versus median not reached; *P* = 0.014; HR = 1.9 [95% CI: 1.1–3.1]). Among 95 patients with a complete morphologic remission (CR), 43 (45%) were MRD-positive by NGS and 52 (55%) were MRD-negative. These MRD-positive CR patients had a shorter overall survival (16.8 months versus median not reached; *P* = 0.013; HR = 2.1 [95% CI: 1.2–3.9]) than did the MRD-negative CR patients. Post-treatment persisting MRD positivity, defined by the same NGS-based test used at diagnosis, is thus a more sensitive biomarker for low-level leukemic clones compared to traditional non-molecular methods and is prognostic of subsequent relapse and death.

## Introduction

Acute myeloid leukemia (AML) is an aggressive clonal hematopoietic stem cell malignancy with a five-year overall survival of ~30%. Although most patients with AML achieve a morphologic complete remission (CR) after intensive induction chemotherapy, approximately half later relapse, with a poor subsequent prognosis. This high rate of relapse implies the post-treatment low-level persistence of residual leukemic clone(s) that were not detected by routine disease monitoring methods. The detection of this post-treatment measurable residual disease (MRD), by a number of different laboratory methods in AML-focused research labs, has been shown to be both a potent prognostic biomarker for long-term disease outcomes (relapse and death) and a shorter-term predictor of early relapse [1–4].

Although MRD is a proven prognostic biomarker in AML, its real-world mass implementation by more oncologists and laboratories is likely curtailed by the requirement for specialized MRD-specific laboratory methods, equipment, and expertize that are difficult to validate, standardize, and implement in a routine clinical diagnostic lab. Flow cytometric assays for AML MRD, for example, are technically complex and difficult to quality control [2, 5]. Single-gene PCR assays for somatic mutations, such as *NPM1*, typically require less technical sophistication but are only applicable to a minority of AML patients, thus leaving most patients without a molecular approach to monitor MRD. Multi-gene NGS-based assays for AML, however, are becoming routinely available in clinical diagnostic labs, for informing targeted therapy and/or risk stratification in pre-treatment AML diagnostic samples.

We hypothesize that the same sensitive clinical laboratory-validated NGS assay, initially applied at the time of the AML diagnosis to define the somatic mutation profile, can also be applied to define molecular MRD after treatment. Utilization of the same clinical diagnostic lab NGS assay both before and after treatment would likely lead to MRD monitoring being applied to more AML patients – by reducing the practical and/or economic barriers associated with the routine clinical diagnostic lab having to send the post-treatment sample to an outside referral lab with appropriate expertise. This local lab in-house serial testing approach could also assist in confirming more NGS-defined somatic mutations as clinically useful MRD markers, and thus further enhance the applicability of these assays to more patients. To assess this hypothesis in a real-world diagnostic lab environment, we utilized the same sensitive clinically validated NGS assay to both define the somatic mutation profile of pre-treatment AML patients and subsequently measure post-treatment MRD – and confirm its prognostic capabilities for long-term clinical outcomes.

## Materials and methods

### Study design and subjects

This was a retrospective, single-center study of 128 patients with a diagnosis of AML as per consensus WHO diagnostic criteria (excluding acute promyelocytic leukemia) [6]. We included all consented Oregon Health & Science University (OHSU) patients from the Beat AML research program, previously described [7], who (1) had serial NGS testing, performed with the same validated clinical assay, at both the time of the initial AML diagnosis (between May-2013 and October-2019) and after the first course of induction chemotherapy; (2) had at least one trackable tier 1 or 2 somatic mutation identified at diagnosis; and (3) had clinical follow-up for a minimum of 6 months from diagnosis or until death. The study was approved by the OHSU institutional review board.

### Post-treatment disease monitoring

Patient response to treatment was evaluated by routine morphologic, cytogenetic, and/or flow cytometric analyses of post-treatment bone marrow samples, as performed in the diagnostic clinical laboratories at OHSU, with previously described methods [8]. Complete remission was defined as the absence of detectable leukemia by all tested methods (bone marrow morphology, cytogenetics/FISH, and flow cytometry) [1]. NGS-based monitoring of the somatic mutations identified at diagnosis was serially performed during the entire time period of disease monitoring (median 6 NGS assays per subject [IQR, 3–7]). For NGS analysis, the diagnostic samples were peripheral blood or bone marrow, and the post-treatment samples were bone marrows. Leukemic relapse was defined per ELN and NCCN guidelines [1, 3] as >5% blasts in the bone marrow, the presence of blasts in the peripheral blood, and/or extramedullary disease.

### NGS-based MRD analysis

High-throughput targeted amplicon-based DNA sequencing was done with a customized NGS assay targeting 42 genes selected for their documented pathogenic role in the initiation or evolution of myeloid malignancies, including acute myeloid leukemia (AML), myelodysplasias, and myeloproliferative neoplasms (supplementary Table S1). From 2013 to December-2017, an AmpliSeq^TM^-based multiplex PCR methodology was used to generate the amplicon-based sequencing libraries that were sequenced on a PGM sequencer (Life Technologies), as previously described [8, 9]. After December-2017, the amplicon-based sequencing libraries were prepared with customized multiplexed QIAseq^TM^ primers, with the addition of unique molecular indices (UMI’s) to facilitate error correction (Qiagen, Hilden, Germany), and sequenced on a NextSeq 500 instrument (Illumina, San Diego, CA). The validated clinical diagnostic lab protocol also includes a supplementary PCR-sizing assay for the common FLT3 internal tandem duplication that, when very large, can be missed by NGS. The customized diagnostic lab validated bioinformatics pipeline has been previously described [8, 9], and utilizes a combination of publically-available and lab-specific tools. The only mutations that were considered as trackable MRD targets for post-treatment monitoring were those that were present in the pre-treatment sample as a pathogenic or likely pathogenic (Tier I or Tier II) somatic mutation as assessed by a board-certified pathologist per consensus tumor NGS interpretation guidelines [10]. For post-treatment samples, NGS reads at genomic coordinates specific for a subject’s unique set of trackable mutations were both manually and computer-inspected for mutant sequencing reads and total sequencing reads [to determine a residual variant allele fraction (VAF)]. The additional manual review step was performed only at loci that had been previously auto-identified by the bioinformatics pipeline as being a somatic pathogenic mutation in the prior diagnostic sample. To assure that the post-treatment mutation signal was above the background sequencing error rate of the assay, the distribution of background errors was determined at each of 798 unique mutational hotspots in a panel of control DNA’s, and a 95% statistical cutoff defined the “limit of blank” at each loci (allowing confident discrimination of low-level mutations from background noise). Using this conservative statistical approach to avoid false positives, at the typical sequencing depth achieved after therapy (average 1900), the 95% lower detection limit of the NGS assay was validated as 0.0024 VAF [8]. Regions with very low background error, such as small insertion-deletions (like the common NPM1 insertion and CEBPA insertion-deletions), had much lower detection limits. Such ultra-low detection limits (below the usual VAF of 0.0024), although theoretically achievable, were never actually observed for any post-treatment indel mutation. The presence or absence of post-treatment MRD was determined by the persistence (significantly above the background in normal samples) of any somatic mutation previously reported in the diagnostic AML sample, with the exception of those in the known preleukemic genes *DNMT3A*, *TET2*, and *ASXL1* (referred hereafter as DTA), as per consensus molecular MRD recommendations [2]. Both versions of this clinical-grade NGS MRD assay (before and after 2017) were validated to stringent CAP and CLIA specifications for licensed patient care use, before and/or after therapy, in a clinical diagnostic laboratory, and cross-validated to each other to confirm equivalent (or improved) analytical operating characteristics. The second-generation assay was also approved for analytical and clinical validity by the Molecular Diagnostic Services (MolDX^®^) Program of the US government’s Centers for Medicare and Medicaid Services (CMS).

### Statistical analysis

Differences in baseline characteristics were assessed with the Wilcoxon rank-sum test for continuous variables and by the *χ*^2^ test for discrete variables. QQ-plots and Kolmogorov-Smirnov tests were used to assess normality of continuous variables. Overall survival (OS) was defined as time from specimen collection for post-treatment NGS testing to death from any cause (or last date known alive). Cumulative incidence of relapse (CIR) (also known as time to relapse) was defined as time from specimen collection for post-treatment NGS testing until relapse, with non-disease death considered a competing event. Kaplan-Meier analysis and log-rank tests were used to compare OS between MRD positive and negative subjects. A Cox proportional hazards analysis was used to evaluate the relationships between the survival outcome and exposure variables. Gray’s test was used to compare CIR between MRD groups. A backward elimination method based on Akaike’s Information Criterion (AIC) was used to find the optimal multivariable analysis model resulting in the lowest prediction error (using the R-software packages: survival, cmprsk, and crrstep). Variables from the univariable analysis with *P* values < 0.1 were eligible to enter the multivariable backward model for selection. All of the variables from the optimal model were used in the final multivariate analysis. All tests were 2-sided, and a *P* value < 0.05 was considered statistically significant. All statistical analyses were performed using SAS statistical software version 9.4 (SAS Institute) and/or R (version 4.2.1).

## Results

### Patient and disease characteristics

One hundred twenty-eight (128) AML patients met the study inclusion criteria, of which 63 (49%) were female. The median (interquartile range [IQR]) age at diagnosis was 58 (44–65) years. Of these 128 subjects, 23 (18%) had secondary (versus de novo) AML, and 45 (35%) had adverse disease prognostic characteristics as per the ELN classification system [11]. Additional patient and disease characteristics are presented in Table 1, with patient-specific details in supplementary Table S2. The median disease monitoring follow-up time after diagnosis was 40 months (95% CI, 34–47), which included a total of 5.5 (mean) samples per subject for serial disease monitoring by NGS.

**Table 1:** AML study patients.

Characteristic	Entire cohort
	(*n* = 128)
Age	
Median (IQR), years	58 (44–65)
Male gender, *n* (%)	65 (51)
de novo AML (versus secondary), *n* (%)	105 (82)
2017 ELN genetic risk, *n* (%)	
Favorable	53 (41)
Intermediate	30 (23)
Adverse	45 (35)
Karyotype, *n* (%)	
Normal	68 (53)
Abnormal	59 (46)
Unknown	1 (0.8)
Mutations at diagnosis (per subject)	
Median (IQR)	3 (2–4)
Mutations at diagnosis, *n* (%)	
* FLT3*/*TP53*/*RUNX1*	61 (48)
* FLT3*	42 (33)
* TP53*	9 (7)
* RUNX1*	15 (12)
* NPM1*	41 (32)
White blood cell count at diagnosis (x10^9^ cells/L)	
Median (IQR)	29 (9–67)
Bone marrow blasts at diagnosis, %	
Median (IQR)	65 (40–90)
7 + 3 Induction chemotherapy, *n* (%)	91 (71)
Stem cell transplant, *n* (%)	65 (51)
Complete remission after induction chemotherapy, *n* (%)	95 (74)

*AML* acute myeloid leukemia, *ELN* European LeukemiaNet, *IQR* interquartile range.

### AML mutation profile

**Fig. 1:**
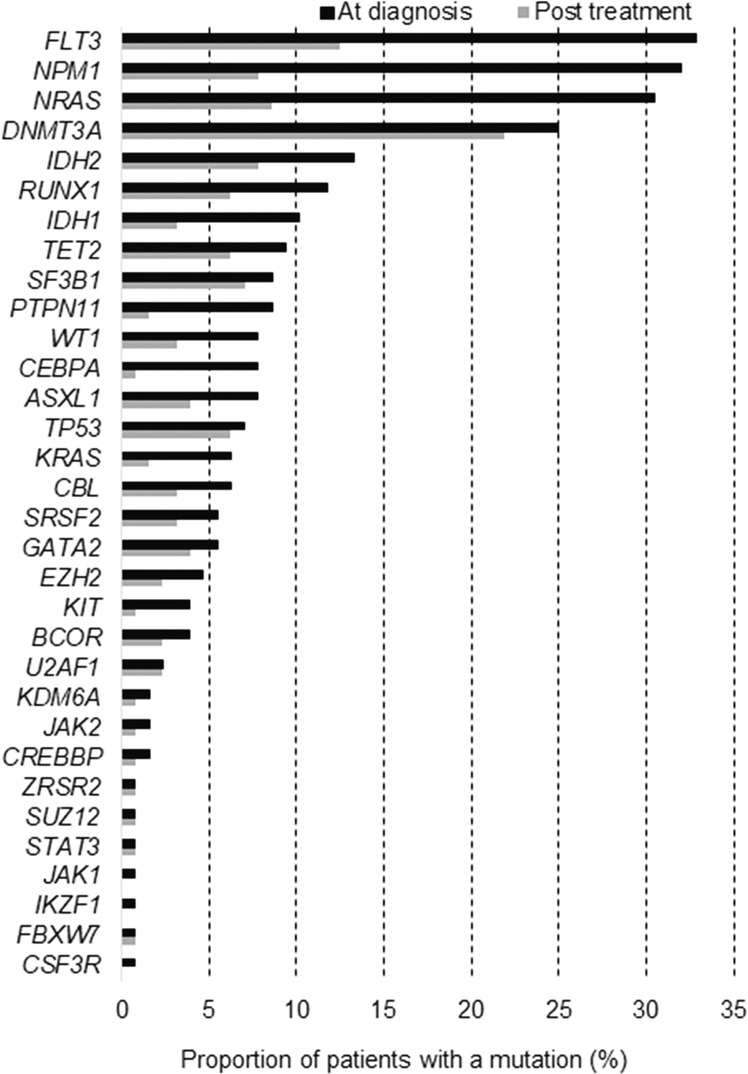
Frequency of somatic gene mutations (by gene) in AML subjects (*n* = 128) at diagnosis and after initial treatment.

**Fig. 2:**
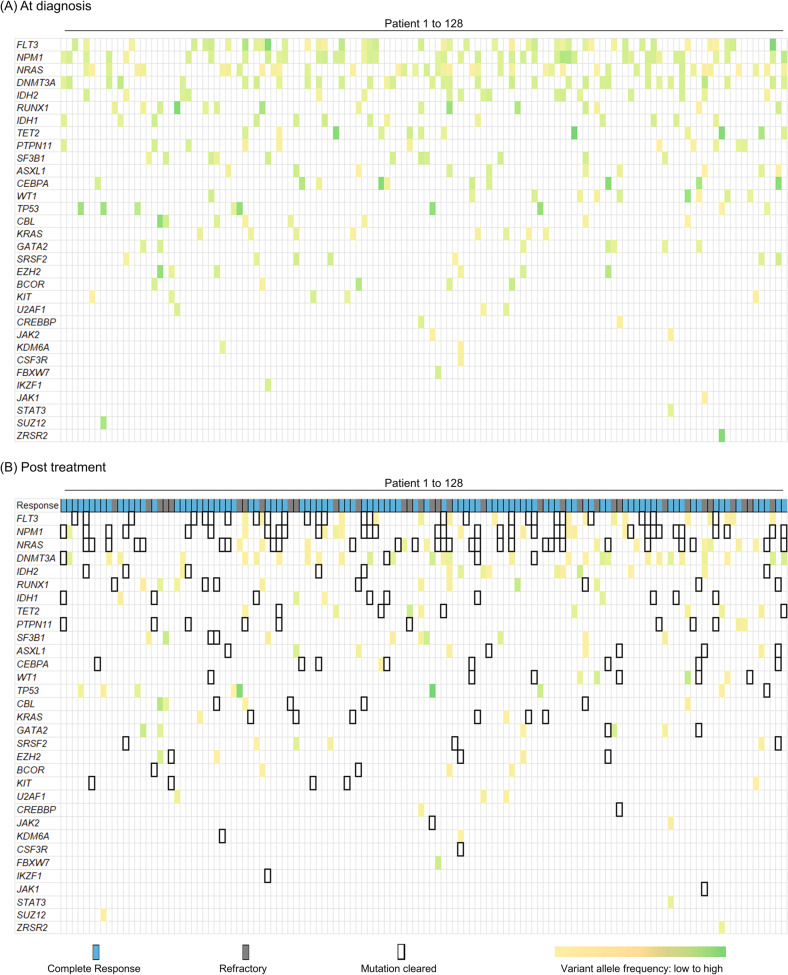
Mutation heat map. Co-mutation profiles of the 128 AML subjects with a somatic mutation at diagnosis (**A**) and after treatment (**B**). Genes are listed in descending order of frequency; the columns represent individual subjects; variant allele frequencies (VAF) are color-coded ranging from 0.985 (green) to 0.009 (yellow), with bordered white boxes representing mutations that became undetectable after treatment. Subjects who achieved a complete response according to routine morphologic, cytogenetic, and/or flow cytometric analyses are depicted as blue in the top row of panel **B**; and subjects who were refractory to treatment are depicted as gray.

At diagnosis, using a 42-gene NGS assay for myeloid malignancies (gene targets detailed in supplementary Table S1), 375 somatic mutations were detected in 32 unique genes. The most frequently mutated genes, in agreement with other large AML studies [12, 13], were *FLT3* (33% of subjects), *NPM1* (32%), *NRAS* (30%), and *DNMT3A* (25%) (Fig. 1). Other genes with mutation frequencies above 10% included *IDH2* (13%), *RUNX1* (12%), and *IDH1* (10%) (Fig. 1). At diagnosis, most patients (76%) carried somatic mutations in more than one gene (mean 2.9 mutations per subject). The median variant allele frequency (VAF) of the somatic AML mutations was 0.40 (IQR 0.19–0.46), confirming a high leukemic disease burden before therapy (Fig. 2A).

### Post-treatment Molecular MRD

The initial induction chemotherapy utilized the standard “7 + 3” regimen (cytarabine plus an anthracycline [daunorubicin or idarubicin]) in 91 subjects (71%) and a variety of different treatments in others (Table 1 and supplementary Table S2). The initial post-induction assessment of treatment response and MRD (by NGS) was performed a median of 32 days (IQR, 28–39) after chemotherapy began, at which time 95 subjects (74%) had achieved a complete remission based on findings from routine bone marrow morphology, flow cytometry, and/or cytogenetics/FISH. To minimize assay-related bias in determining post-treatment molecular MRD, the NGS-based MRD assay that was performed after treatment, with a detection limit of 0.0024 VAF [8], utilized the exact same clinical laboratory-validated protocol and bioinformatics analysis as the prior NGS performed on the diagnostic AML sample (with the addition of a manual inspection at all trackable mutation loci.) This initial post-treatment NGS assay detected the residual presence of at least one somatic tier 1 or 2 mutation previously seen in the diagnostic sample in 81 (63%) subjects. Of these 81 subjects, 12 carried detectable residual somatic mutations after therapy only in the “preleukemic” genes *DNMT3A*, *TET2*, and/or *ASXL1* (DTA); mutations in these genes are commonly found in benign age-related clonal hematopoiesis, often persist after therapy without prognostic significance, and have been recommended by consensus experts to be excluded as reliable molecular MRD markers [2, 3, 14, 15]. Of the 69 (54%) subjects ultimately classified as MRD-positive (excluding persisting mutations in DTA genes), there were 115 residual mutations in 26 unique genes (median VAF = 0.064, IQR 0.016–0.24). In comparison, 53% of the mutations present at diagnosis became undetectable after treatment, with 59 (46%) MRD-negative subjects, each having no detectable post-treatment mutation in any non-DTA gene. Figure 2 depicts the comparative pre- and post-treatment mutation burdens for all 375 trackable somatic mutations. The mutations that most frequently persisted after therapy were in *DNMT3A* (28 subjects, but not coded as MRD-positive), *FLT3* (16 subjects), *NRAS* (11 subjects), *IDH2* (10 subjects), and *NPM1* (10 subjects) (Figs. 1 and 2). More than half of the 69 MRD-positive subjects (*n* = 43; 62%) had achieved a complete remission (no detectable leukemia cells) with routine hematopathology testing (supplementary Table S3), confirming the enhanced analytical sensitivity of the NGS-based MRD method for detecting low-level persistent disease.

### Clinical outcomes

The post-induction management of this AML cohort was heterogeneous and included allogeneic hematopoietic stem cell transplantation in 51% of the subjects (*n* = 65) (Table 1). The median disease monitoring follow-up time after the initial post-induction MRD assessment was 39 months (95% CI, 33–44), during which time 60 (47%) subjects died. The median time to death among these 60 subjects was 8.5 months (IQR 4.5–16), with 90% of the deaths occurring before 20 months. Despite patient-specific optimized therapies, relapsed AML occurred in 63 subjects (49%), a median of 8.0 months (IQR, 4.4–12) after the initial post-induction MRD assessment. Of these relapsed subjects, 75% (*n* = 47) subsequently died, a median of 1.4 months after relapse (IQR, 0–5.4). The other 13 patients died of non-leukemic causes, typically infection or GVHD resulting from their aggressive treatments (median 8.2 months after induction, IQR 3.5–14). The disease characteristics that were expectedly prognostic of poor long-term outcomes (earlier relapse and death) included ELN risk category, a diagnosis of secondary (versus de novo) AML, and the presence of a mutation in the known poor-prognosis genes *TP53*, *RUNX1*, or *FLT3* (Table 2). The treatments that led to superior long-term outcomes included the use of 7 + 3 induction chemotherapy (versus other less intensive induction regimens) and consolidation with a hematopoietic stem cell transplant (Table 2). Each of these proven prognostic factors in this single-institution AML cohort has also been shown to be prognostic in larger multi-site AML studies, confirming the similarity of the disease and treatment parameters in the AML subjects in this study group as compared to other large AML cohorts.

**Table 2:** Univariable risk factors for relapse and death.

Clinical Characteristic	Cumulative Incidence of Relapse		Overall Survival	
(*n* = 128 AML subjects)	(*n* = 63 relapses)		(*n* = 60 deaths)	
	Hazard ratio (95% CI)	*P* value	Hazard ratio (95% CI)	*P* value
Age, per year	1.01 (0.99–1.03)	0.39	1.01 (0.99–1.03)	0.27
Male (vs female) gender	0.93 (0.57–1.52)	0.77	1.36 (0.81–2.26)	0.24
**Secondary (vs de novo) AML**	**2.51 (1.41–4.47)**	**0.002**	**2.13 (1.19–3.83)**	**0.01**
**ELN risk (adverse/intermediate vs favorable)**	**1.84 (1.12–3.03)**	**0.02**	**3.58 (1.93–6.63)**	**<0.001**
Karyotype (abnormal vs normal)	1.30 (0.79–2.13)	0.30	0.98 (0.59–1.63)	0.93
Mutations at diagnosis (per mutation)	1.05 (0.90–1.22)	0.51	1.12 (0.96–1.29)	0.14
***FLT3*/*TP53*/*RUNX1*** **mutation**	**1.57 (0.97–2.57)**	**0.07**	**2.84 (1.66–4.86)**	**<0.001**
*NPM1* mutation (detected vs not detected)	0.83 (0.49–1.40)	0.48	0.84 (0.48–1.45)	0.52
White blood cell count (per 10^9^ cells/L)	1.00 (1.00–1.01)	0.15	1.00 (1.00–1.01)	0.17
Bone marrow blasts (per each %)	1.00 (0.99–1.01)	0.44	1.00 (0.99–1.01)	0.96
**7** + **3 Induction chemotherapy (yes vs no)**	**0.65 (0.40–1.07)**	**0.09**	**0.61 (0.36–1.03)**	**0.07**
**Stem cell transplant (yes vs no)**	**0.52 (0.32–0.84)**	**0.008**	**0.54 (0.32–0.90)**	**0.02**
**MRD (by NGS; positive vs negative)**	**1.88 (1.14–3.10)**	**0.01**	**2.18 (1.27–3.75)**	**0.005**

*AML* acute myeloid leukemia, *ELN* European LeukemiaNet.

BOLDED variables (6) qualified for the multivariable analyses.

### Persisting molecular MRD predicts worse long-term clinical outcomes

**Fig. 3:**
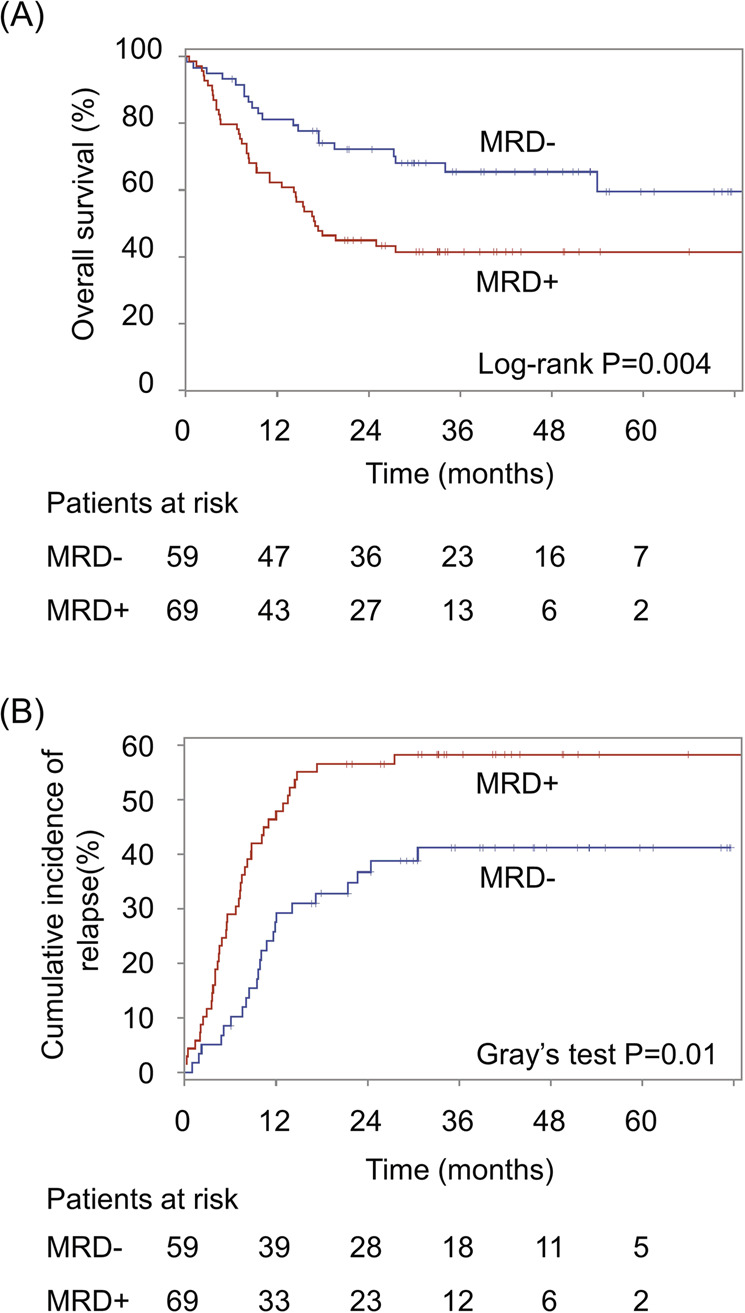
Survival analyses. **A** Overall survival in MRD-positive (by NGS) vs MRD-negative subjects. **B** Time to relapse (cumulative incidence of relapse) in MRD-positive (by NGS) vs MRD-negative subjects, controlling for the competing risk of non-relapse mortality. The number of patients at risk (i.e., had no event and were not censored before the time point) is shown below each survival graph.

The 69 subjects who had a persisting detectable non-DTA mutation after their initial induction chemotherapy (MRD-pos by NGS) had a significantly shorter overall survival (median 17 months) compared to the 59 subjects who cleared each of their known non-DTA pre-treatment somatic mutations [median survival not reached; log-rank *P* = 0.0036; hazard ratio for death = 2.2 (95% CI 1.3–3.7)] (Fig. 3A). After controlling for the competing risk of non-leukemic mortality (in 13 subjects), these 69 MRD-positive subjects also had a significantly shorter time to relapse (median 13.6 months) than the MRD-negative subjects [median time to relapse not reached; Gray’s *P* = 0.014; hazard ratio = 1.9 (95% CI, 1.1–3.1)] (Fig. 3B). To assess whether the poor prognosis imparted by having a persistently detectable post-induction mutation (MRD) was independent of other prognostic factors, all of the 6 variables in Table 2 that were associated with a significantly increased univariate risk for relapse and death were included in a multivariable analysis. This initial 6-variable analysis included secondary AML, ELN risk category, FLT3/TP53/RUNX1 mutation, the use of intensive 7 + 3 induction chemotherapy, stem cell transplant, and MRD detected by NGS. In the final models, ELN and 7 + 3 were excluded as independent predictors of relapse (both P > 0.2). After adjustment for secondary AML, *FLT3*/*TP53*/*RUNX1* mutations, and stem cell transplant, the presence of MRD, as assessed by a post-induction persisting NGS-detectable mutation, was independently prognostic of both relapse (HR = 1.85; 95% CI 1.07–3.20; *P* = 0.027) and death (HR = 2.04; 95% CI 1.17–3.57; *P* = 0.013) (Table 3). Furthermore, this significant independent excess risk imparted by persisting post-treatment NGS-positivity was of quantitatively similar magnitude to the 2- to 3-fold excess risk imparted by each of the 3 other more traditional independent risk factors for poor prognosis (Table 3).

**Table 3:** Multivariable risk factors for relapse and death.

Variables Included^a^	*N*	Events			Cumulative incidence of relapse		Overall survival	
		Relapse	Relapse-related death	Non-relapse death	HR (95% CI)	*P* value	HR (95% CI)	*P* value
MRD-positive by NGS	69	40	34	6	1.85 (1.07–3.20)	0.027	2.04 (1.17–3.57)	0.013
Secondary AML (not de novo)	23	17	13	2	2.40 (1.36–4.25)	0.003	1.78 (0.97–3.26)	0.062
*FLT3*, *TP53*, *RUNX1* mutation	61	34	31	9	1.78 (1.08–2.94)	0.024	2.93 (1.70–5.06)	<0.001
Stem cell transplant	65	26	15	10	0.39 (0.23–0.66)	<0.001	0.46 (0.27–0.77)	0.004

NGS indicates a 42-gene next-generation sequencing assay.

^a^ELN & 7 + 3 induction chemotherapy not included in the final 4-variable multivariable model; deselected by backward elimination method (both with *P* > 0.2 for relapse)

### NGS is a better and more sensitive prognostic MRD method compared to traditional non-molecular methods

**Fig. 4:**
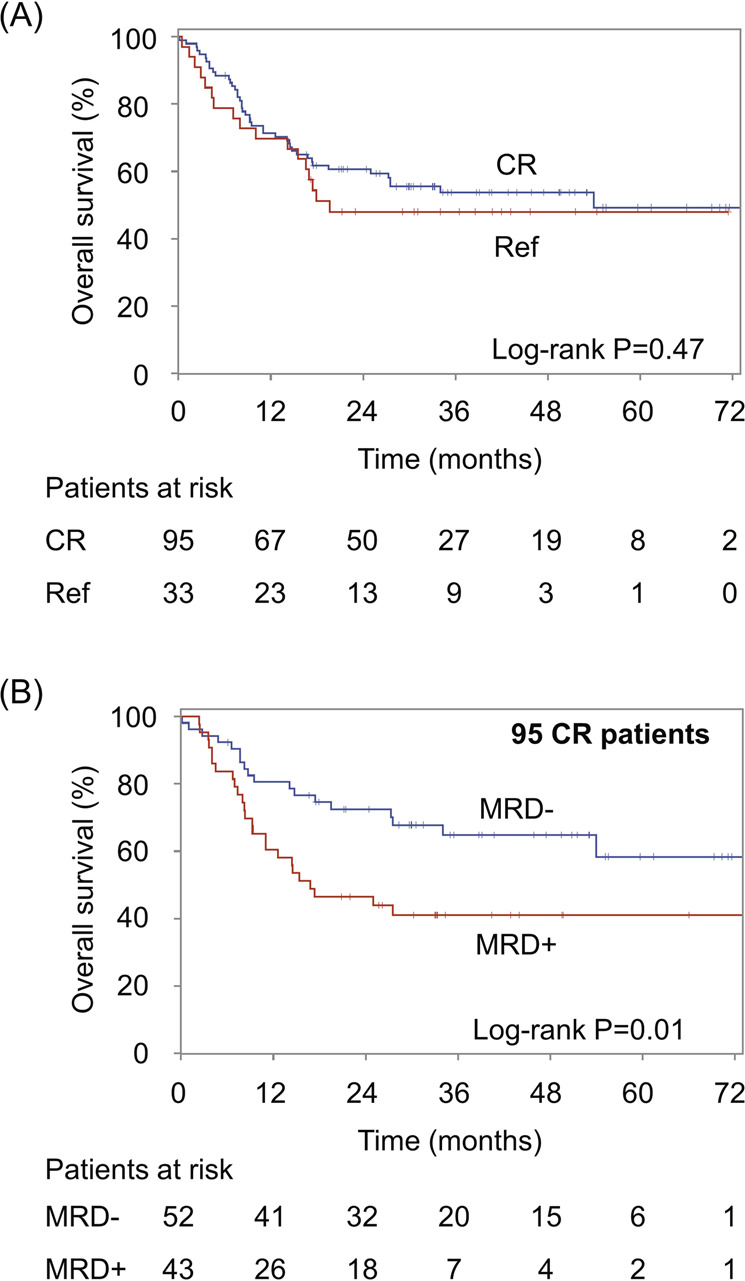
Survival analyses. **A** Overall survival in subjects who were complete responders (CR) (by non-molecular methods) vs those refractory (Ref) to the initial treatment. **B** Overall survival in MRD-positive (by NGS) vs MRD-negative subjects, among only the 95 CR patients. The number of patients at risk (i.e., had no event and were not censored before the time point) is shown below each survival graph.

Of the 95 AML subjects who achieved a post-induction complete remission by standard hematopathologic testing (morphology, flow cytometry, and cytogenetics/FISH), 43 (45%) subsequently died. In comparison, of the 33 subjects with treatment-refractory disease (by the same methods), 17 (52%) subsequently died (supplementary Table S3). Overall survival did not significantly differ between those with complete remission based on these standards measures versus those refractory to the initial induction therapy (log-rank *P* = 0.47) (Fig. 4A). The high mortality rate in the 95 subjects with a presumed “complete” treatment response (by traditional non-molecular methods) is likely due to the presence of persisting mutation-positive AML clones in 43 (45%) of these subjects by more sensitive NGS-based methods. In support of this hypothesis, among only these 95 subjects with a complete remission (CR) after initial induction chemotherapy, the overall survival was significantly shorter among the 43 NGS-MRD-positive CR subjects (median 17 months) than among the 52 NGS-MRD-negative CR subjects [median survival not reached; log-rank *P* = 0.013; hazard ratio 2.1 (95% CI, 1.2–3.9)] (Fig. 4B).

## Discussion

In this retrospective study of 128 AML patients who were tested by the same sensitive 42-gene targeted NGS panel both at diagnosis and after their initial induction chemotherapy, the post-treatment persistence of a detectable somatic mutation (MRD-positivity) was shown to be independently predictive of a significantly inferior clinical outcome (earlier relapse and death). The assessment of post-induction MRD by an NGS-based method that was universally applied to all pre-and post-treatment AML samples in a routine diagnostic pathology lab was also shown to be more analytically sensitive than traditional non-molecular MRD-detection methodologies such as morphology, flow cytometry, and cytogenetics/FISH. In particular, only 26% of the post-treatment AML subjects were MRD-positive by these traditional diagnostic methods (failing to achieve a traditional “complete remission”), compared to 54% who were MRD-positive by NGS. Furthermore, this significant increase in MRD diagnostic yield afforded by NGS was not just indicative of an analytically superior laboratory detection methodology for post-treatment persisting clonal disease, but was also shown to be a clinically relevant prognostic biomarker for predicting long-term disease outcomes, as confirmed by the significantly shorter overall survival among the 45% of CR subjects who were NGS-positive (as compared to those CR subjects with no persisting mutations). Conversely, the attainment of a traditional non-molecular CR was not prognostic of an improved overall survival, likely due to the persistence of low-level post-treatment AML clones detectable only by NGS. The detection of post-treatment MRD by this more sensitive NGS method (in more than double the number of subjects detectable by non-molecular methods) thus defines a routinely available laboratory test to identify more AML patients with low-level residual disease who are most likely to relapse and die. Identifying these high-risk patients is a necessary first step toward applying more intensive induction and/or consolidation-based therapies towards the ultimate therapeutic goal of disease eradication (to impart the best long-term prognosis). Perhaps equally important, however, is the clinical utility of this same sensitive NGS MRD method to identify the ~50% of AML patients who are at lower risk after their initial therapy and may not require additional (often toxic) treatments to achieve improved clinical outcomes, beyond standard of care consolidation (with or without stem cell transplant).

The NGS-based assay approach that was used in this study was novel in that the same clinically validated method targeting 42 gene targets was routinely applied to all pre- and post-treatment samples [extending 40 months and 6 samples per subject (median) after diagnosis] within a conventional diagnostic clinical pathology laboratory. Although this “all time point” universal NGS assay did require a manual inspection of NGS reads for low-level trackable mutations (after treatment), and an initial extensive validation phase to meet stringent US government regulatory requirements for a clinical diagnostic test, there was no need for additional dedicated post-treatment MRD-specific methods, requiring specialized reagents, equipment, and/or bioinformatics. This practical approach to MRD assay validation may allow analogous molecular MRD assays to become more universally available in routine diagnostic clinical labs, thus improving patient access to these proven life-extending biomarkers, beyond the more restricted and specialized research lab settings where such technologies have been traditionally available.

Our NGS-based MRD assay that could be practically and equivalently applied to all pre- and post-treatment AML samples was not only more analytically sensitive and prognostically relevant than the non-molecular assays traditionally used to assess disease burdens, but it was also more clinically applicable. In particular, 85% of our AML patients had at least one somatic non-DTA mutation that could be monitored by post-treatment clinical-grade NGS (relative to research-based exome sequencing) (unpublished internal data). The percentage of mutation-negative AML cases not eligible for NGS-based disease monitoring will, of course, be dependent on the number of genes being targeted by the NGS assay. However, when designing NGS assays, most clinical laboratories must appropriately balance the inherent desire to target more genes with the ensuing inevitable disadvantage of requiring more overall sequence depth to achieve a sufficiently low mutant allele detection limit - with a consequent increase in cost and turnaround time. The 42-gene NGS panel used in this study was large enough to detect an NGS-trackable mutation in 85% of AML patients, but small enough to achieve sufficiently deep sequencing coverage to confer a low-level detection limit below 0.003 VAF.

The poor prognosis imparted by persisting post-treatment NGS-detectable MRD has also been reported by other investigators, at varying AML post-treatment time points, including after initial induction chemotherapy [14, 16], before consolidation with stem cell transplantation [8, 17], and after stem cell transplantation [18]. Other non-NGS methods for MRD detection, including flow cytometry and cytogenetics/FISH, have also been consistently shown to predict poor subsequent AML outcomes [4]. However, as we have shown, these less-sensitive methods may yield false-negative MRD results in a significant fraction of patients. Given the current lack of standardization of AML NGS assays in different labs [19], the specific reagent and bioinformatic technical details of these other comparable MRD studies were expectedly quite heterogeneous. In contrast, our assay used a standardized NGS protocol that was thoroughly validated, by rigid government-mandated regulatory guidelines, for routine diagnostic use in a licensed clinical laboratory. More widespread clinical deployment of standardized NGS assays, in routine clinical diagnostic labs that provide other conventional leukemia diagnostic services, will be necessary to achieve the long-term goal of making these life-extending assays available to a higher proportion of AML patients.

The complexity and non-standardization of MRD assessments in AML stems from not only the choice of methodology (flow cytometry, RT-PCR, FISH, NGS) but also the choice of gene/protein targets. For NGS-based MRD assays, which typically target dozens of myeloid malignancy-related genes, there is abundant evidence confirming that not all genes and/or mutations are equivalently useful for MRD prognosis. For example, some mutations, most notably the common *NPM1* C-terminal insertion, have been well documented to impart a significant subsequent relapse risk when they persist after AML therapy [1, 2, 15]. Alternatively, other common AML mutations that persist after treatment do not impart substantial additional relapse risk. These include mutations in the *DNMT3A*, *TET2*, and *ASXL1* (DTA) genes, which typically originate in the early preleukemic and/or clonal hematopoiesis stage of disease [2, 14, 18, 20, 21]. The inability of these DTA mutations to function as reliable MRD markers for predicting disease outcomes has led to consensus recommendations from both the ELN and NCCN to specifically exclude the DTA genes when assessing molecular MRD by NGS-based methods [1–3, 15]. We have followed these consensus guidelines and have therefore specifically counted only those persisting post-induction mutations in non-DTA genes as being “MRD-positive.” In addition, all of the 39 non-DTA genes assessed in this study were assumed to be quantitatively equivalent MRD risk factors in our outcomes-based analyses. Although this equivalent risk assumption is undoubtedly over-simplistic, given the biochemical heterogeneity of the affected mutations, a more sophisticated model would require gene-specific quantitative risk data that could only be generated from comprehensive genomic and clinical outcomes data aggregated from thousands of AML patients – which is not yet available.

Relapsed AML is often clonally evolved compared to the pre-treatment precursor clone(s), frequently with the addition of new or expanding mutations, beyond those initially seen at diagnosis. These newly evolved or expanding mutations will not be detectable at low clonal burdens (<0.02 VAF) before frank relapse using our “trackable mutation” strategy for MRD monitoring, a specific limitation of this study. The median clonal mutation burden after treatment, however (0.064 VAF) will be easily detectable by NGS, without any pre-test knowledge of its prior presence. Other limitations of this study include its relatively small size, restriction of MRD monitoring to a single post-treatment time point, and the single-site retrospective patient cohort. Potential selection bias in the AML study subjects, always a concern in retrospective studies, has been minimized by including all AML patients at our institution with serial NGS monitoring who consented to participate in the larger Beat AML study, which has done extensive genomic and drug susceptibility characterizations of this disease [7, 22]. The mutation profile, prognostic risk factors, and demographics of the resulting 128 AML subjects analyzed in this study are thus not significantly different than either the larger Beat AML patient population (most without serial NGS monitoring) or other large AML cohorts from other studies [7, 13, 22].

The vast majority of patients in studies assessing the prognostic value of MRD, including ours, were treated with intensive chemotherapy modalities, typically including an initial induction with anthracyclines and cytarabine (the standard “7 + 3” therapy), a therapeutic approach that has not substantively changed in decades. In elderly patients and/or those deemed unfit to tolerate this traditional intensive chemotherapy regimen, an exciting new treatment option is the BCL-2 inhibitor venetoclax, which has shown good tolerability, high response rates, and prolonged overall survival when combined with hypomethylating agents or low dose cytarabine [23, 24]. An important unanswered question in venetoclax-treated patients is whether achievement of MRD-negativity, which does occur in a substantial minority of these patients, is as strong of a prognostic marker for long-term outcomes as it is in patients treated with intensive 7 + 3 based regimens. Preliminary flow cytometric MRD determinations in the original venetoclax-based clinical trials suggested that MRD-negativity achieved after this less intensive treatment did indeed portend a significantly improved prognosis [25, 26]. Future studies in these less intensively treated patients, with more sensitive and clinically applicable NGS-based MRD methods, will be required to definitively address the important question as to whether the prognostic capabilities of MRD can be generically extended to a wider heterogeneity of treatments.

In summary, we have shown that in newly diagnosed AML subjects initially treated with intensive induction chemotherapy, the attainment of an NGS-defined absence of detectable molecular MRD at the end of the induction phase is a significant and independent prognostic marker for subsequent relapse and death. The increasing availability of these sensitive MRD assays in a growing number of clinical diagnostic labs should allow the identification of the significant fraction of patients (45% in our cohort) with low-level persisting disease (and its associated poorer outcomes), even after achieving a complete remission by less sensitive laboratory methods. Given this valuable prognostic information, future clinical trials should assess whether more intensive therapies directed specifically at MRD-positive patients can improve long-term outcomes without excessive toxicity. For example, in AML patients with an initial favorable-risk genetic profile who remain MRD-positive after intensive induction chemotherapy, would subsequent stem cell transplantation ameliorate the relapse risk? MRD analyses are also now being used in clinical trials of novel induction and/or consolidation therapies as a surrogate endpoint for longer-term relapse and death – to hopefully accelerate the current suboptimal decade-long drug development timeline. These highly sensitive NGS assays will be a crucial tool for defining the ultimate goal for patients undergoing treatment for this deadly malignancy, namely disease eradication.

## Supplementary information

Supplementary Tables (3) with Legend

## Data Availability

All data generated or analyzed during this study are included in this published article [and its supplementary information files].
