# Supplementary material for: NGS-defined measurable residual disease (MRD) after initial chemotherapy as a prognostic biomarker for acute myeloid leukemia
Source: Blood Cancer J. 2023 Apr 24;13(1):59. doi: 10.1038/s41408-023-00833-7 (PMC10123056; doi:10.1038/s41408-023-00833-7)
Supplement: Supplementary file 1 — Supplementary Tables (3) with Legend [file 41408_2023_833_MOESM1_ESM.pdf]

Supplementary Information:

Supplementary Table S1:

Excel file listing the 42 genes (and target regions) covered by the NGS assay used for diagnosis and MRD monitoring of AML subjects

Supplementary Table S2:

Excel file listing the demographic characteristics, risk factors, MRD status, and clinical outcomes of each of the 128 AML subjects

Supplementary Table S3:

Excel file (two-by-two table) detailing the number of subjects with and without a complete remission (by morphologic, cytogenetic, or flow cytometric analyses) versus those with and without NGS-defined MRD.

Table S1. Names and target regions of the genes sequenced by NGS

**Gene**	**Coverage**
ABL1	selected hotspot exons
ASXL1	all coding exons
BCOR	all coding exons
CBL	selected hotspot exons
CBLB	selected hotspot exons
CEBPA	all coding exons
CREBBP	all coding exons
CSF3R	selected hotspot exons
DNMT3A	all coding exons
ETV6	all coding exons
EZH2	all coding exons
FBXW7	all coding exons
FLT3	selected hotspot exons
GATA1	all coding exons
GATA2	selected hotspot exons
HRAS	selected hotspot exons
IDH1	selected hotspot exons
IDH2	selected hotspot exons
IKZF1	all coding exons
IL7R	selected hotspot exons
JAK1	selected hotspot exons
JAK2	selected hotspot exons
JAK3	selected hotspot exons
KDM6A/UTX	all coding exons
KIT	selected hotspot exons
KRAS	selected hotspot exons
MPL	selected hotspot exons
NOTCH1	selected hotspot exons
NPM1	selected hotspot exons
NRAS	selected hotspot exons
PAX5	all coding exons
PTPN11 (SHP2)	selected hotspot exons
RUNX1	all coding exons
SF3B1	all coding exons
SRSF2	all coding exons
STAT3	selected hotspot exons
SUZ12	all coding exons
TET2	all coding exons
TP53	all coding exons
U2AF1 (U2AF35)	all coding exons
WT1	all coding exons
ZRSR2	all coding exons

Table S2. Patient-specific characteristics

Patient number	MRD (by NGS)	Complete Remission	Overall Survival (days)	Vital Status	Days to Relapse/Death	Competing Risk (2=Competing Event)	Age	Sex	deNovo AML	2017 ELN_Risk	FLT3/TP53/ RUNX1	7+3 Induction	Stem cell transplant
Q001	MRD-Neg	CR	2138	Alive	2138	0	60	F	Yes	Favorable	0	Yes	No
Q002	MRD-Pos	CR	2309	Alive	2309	0	66	M	Yes	Favorable	0	Yes	No
Q003	MRD-Neg	CR	233	Dead	110	1	30	F	No	Intermed	1	Yes	No
Q004	MRD-Pos	CR	211	Dead	169	1	63	M	No	Adverse	1	Yes	Yes
Q005	MRD-Neg	CR	2340	Alive	819	1	57	M	Yes	Favorable	1	Yes	No
Q006	MRD-Neg	CR	1816	Alive	1816	0	35	F	Yes	Favorable	0	Yes	No
Q007	MRD-Neg	CR	2178	Alive	2178	0	55	M	Yes	Favorable	0	No	Yes
Q008	MRD-Pos	CR	252	Dead	114	1	71	F	Yes	Adverse	1	Yes	No
Q009	MRD-Neg	CR	2162	Alive	2162	0	63	F	Yes	Favorable	0	Yes	Yes
Q010	MRD-Neg	Ref	2172	Alive	2172	0	62	M	No	Adverse	1	No	Yes
Q011	MRD-Pos	CR	2008	Alive	2008	0	62	M	Yes	Intermed	0	Yes	Yes
Q012	MRD-Neg	CR	2108	Alive	526	1	69	M	Yes	Favorable	0	No	Yes
Q013	MRD-Neg	CR	1641	Dead	1641	2	48	F	Yes	Intermed	1	Yes	Yes
Q014	MRD-Pos	CR	123	Dead	123	1	62	F	Yes	Adverse	1	Yes	No
Q015	MRD-Pos	CR	334	Dead	334	2	68	M	Yes	Adverse	1	No	Yes
Q016	MRD-Pos	Ref	472	Dead	199	1	36	F	Yes	Adverse	0	Yes	Yes
Q017	MRD-Neg	CR	430	Dead	430	2	71	F	Yes	Intermed	0	Yes	Yes
Q018	MRD-Pos	Ref	216	Dead	216	1	45	M	No	Adverse	1	Yes	No
Q019	MRD-Pos	Ref	64	Dead	64	1	68	M	No	Intermed	0	No	No
Q020	MRD-Neg	Ref	1313	Alive	1313	0	37	M	Yes	Favorable	0	Yes	Yes
Q021	MRD-Pos	CR	205	Dead	205	2	57	M	Yes	Adverse	1	Yes	Yes
Q022	MRD-Pos	CR	634	Alive	420	1	65	M	Yes	Intermed	0	Yes	No
Q023	MRD-Neg	CR	3	Dead	3	2	42	M	Yes	Favorable	0	No	No
Q024	MRD-Neg	CR	1869	Alive	1869	0	29	F	Yes	Intermed	1	Yes	Yes
Q025	MRD-Pos	CR	1505	Alive	1505	0	64	M	Yes	Favorable	0	No	No
Q026	MRD-Neg	CR	199	Dead	68	1	43	F	Yes	Adverse	1	No	No
Q027	MRD-Neg	CR	836	Dead	735	1	60	M	Yes	Favorable	1	No	No
Q028	MRD-Pos	CR	468	Dead	365	1	76	M	Yes	Adverse	1	No	No
Q029	MRD-Neg	CR	1035	Dead	1011	1	29	M	Yes	Adverse	0	No	Yes
Q030	MRD-Neg	CR	290	Dead	290	2	62	F	Yes	Adverse	1	Yes	Yes
Q031	MRD-Pos	CR	244	Dead	228	1	68	F	Yes	Adverse	1	Yes	Yes
Q032	MRD-Pos	Ref	598	Dead	440	1	51	M	Yes	Adverse	1	No	Yes
Q033	MRD-Pos	Ref	132	Dead	132	1	53	M	Yes	Intermed	1	Yes	No
Q034	MRD-Neg	CR	1689	Alive	477	1	51	F	Yes	Favorable	0	Yes	Yes
Q035	MRD-Pos	CR	1510	Alive	1510	0	61	M	Yes	Intermed	1	Yes	Yes
Q036	MRD-Pos	Ref	1653	Alive	1653	0	66	F	Yes	Adverse	1	Yes	Yes
Q037	MRD-Pos	CR	836	Dead	836	2	68	M	Yes	Intermed	1	Yes	Yes
Q038	MRD-Neg	CR	1677	Alive	1677	0	35	F	Yes	Favorable	0	Yes	No
Q039	MRD-Neg	CR	1078	Alive	1078	0	61	M	Yes	Favorable	0	Yes	No
Q040	MRD-Neg	CR	1614	Alive	1614	0	53	F	Yes	Favorable	1	No	No
Q041	MRD-Neg	Ref	524	Alive	524	0	36	M	Yes	Favorable	0	Yes	No
Q042	MRD-Pos	Ref	88	Dead	88	1	68	M	No	Adverse	0	Yes	No
Q043	MRD-Neg	CR	1611	Alive	1611	0	40	M	Yes	Favorable	0	Yes	Yes
Q044	MRD-Pos	CR	72	Dead	63	1	53	F	Yes	Intermed	1	No	No
Q045	MRD-Neg	CR	593	Dead	294	1	64	M	Yes	Favorable	0	No	Yes
Q046	MRD-Neg	CR	1567	Alive	1567	0	65	F	Yes	Intermed	1	Yes	Yes
Q047	MRD-Pos	CR	760	Dead	241	1	24	F	No	Intermed	1	Yes	No
Q048	MRD-Pos	CR	529	Dead	529	1	52	F	Yes	Intermed	0	Yes	No
Q049	MRD-Pos	Ref	1569	Alive	1569	0	61	F	Yes	Favorable	0	Yes	Yes
Q050	MRD-Pos	CR	283	Dead	9	1	44	F	Yes	Intermed	1	Yes	Yes
Q051	MRD-Neg	CR	1504	Alive	301	1	36	F	Yes	Favorable	0	Yes	Yes
Q052	MRD-Neg	CR	1544	Alive	230	1	58	F	No	Favorable	0	Yes	Yes
Q053	MRD-Neg	Ref	531	Dead	531	2	62	M	Yes	Adverse	1	Yes	No
Q054	MRD-Neg	CR	930	Alive	930	0	51	F	Yes	Favorable	1	Yes	No
Q056	MRD-Neg	CR	829	Dead	246	1	67	M	Yes	Favorable	1	Yes	Yes
Q057	MRD-Pos	CR	110	Dead	110	1	65	F	Yes	Intermed	1	Yes	No
Q058	MRD-Pos	CR	1014	Alive	1014	0	46	F	Yes	Favorable	0	Yes	No
Q059	MRD-Neg	CR	1442	Alive	1442	0	50	F	Yes	Adverse	0	Yes	Yes
Q060	MRD-Pos	CR	1336	Alive	1336	0	75	M	Yes	Intermed	0	Yes	No
Q061	MRD-Neg	CR	1395	Alive	1395	0	34	F	Yes	Favorable	0	Yes	No
Q063	MRD-Pos	Ref	244	Dead	244	1	34	M	Yes	Adverse	0	No	No
Q064	MRD-Neg	Ref	1389	Alive	1389	0	26	F	Yes	Adverse	0	No	Yes
Q065	MRD-Neg	CR	31	Dead	31	2	29	M	No	Intermed	0	No	No
Q066	MRD-Pos	Ref	503	Dead	171	1	69	F	Yes	Adverse	1	Yes	No
Q067	MRD-Pos	CR	1302	Alive	1302	0	58	F	No	Adverse	0	No	Yes
Q068	MRD-Pos	Ref	431	Dead	413	1	67	M	No	Adverse	1	Yes	Yes
Q069	MRD-Pos	Ref	1276	Alive	1276	0	60	F	Yes	Favorable	0	Yes	Yes
Q070	MRD-Neg	CR	531	Dead	354	1	52	F	Yes	Favorable	1	Yes	No
Q071	MRD-Pos	Ref	1240	Alive	1240	0	56	F	No	Adverse	1	Yes	Yes
Q072	MRD-Neg	CR	1177	Alive	1177	0	64	M	Yes	Intermed	0	Yes	Yes
Q073	MRD-Pos	CR	512	Dead	448	1	81	M	Yes	Favorable	0	No	No
Q074	MRD-Neg	CR	742	Alive	742	0	69	F	Yes	Favorable	1	No	No
Q076	MRD-Neg	CR	185	Alive	185	0	47	M	Yes	Favorable	0	Yes	No
Q077	MRD-Neg	CR	1189	Alive	1189	0	38	F	Yes	Favorable	0	Yes	No
Q078	MRD-Pos	Ref	1172	Alive	149	1	72	F	Yes	Adverse	0	No	No
Q081	MRD-Pos	CR	250	Dead	250	2	61	M	Yes	Favorable	0	No	Yes
Q082	MRD-Pos	CR	1011	Alive	1011	0	66	M	Yes	Adverse	1	Yes	Yes
Q083	MRD-Pos	CR	1045	Alive	1045	0	60	M	Yes	Favorable	0	Yes	Yes
Q084	MRD-Pos	CR	281	Dead	65	1	21	M	Yes	Adverse	0	Yes	No
Q085	MRD-Pos	CR	335	Dead	165	1	36	F	Yes	Favorable	1	No	Yes
Q086	MRD-Pos	CR	1004	Alive	644	1	39	F	Yes	Adverse	1	Yes	Yes
Q087	MRD-Pos	CR	1006	Alive	902	1	23	F	Yes	Favorable	0	Yes	No
Q088	MRD-Neg	CR	264	Dead	260	1	22	M	Yes	Adverse	1	No	Yes

Patient number	MRD (by NGS)	Complete Remission	Overall Survival (days)	Vital Status	Days to Relapse/Death	Competing Risk (2=Competing Event)	Age	Sex	deNovo AML	2017 ELN_Risk	FLT3/TP53/ RUNX1	7+3 Induction	Stem cell transplant
Q089	MRD-Neg	CR	233	Dead	156	1	55	M	Yes	Intermed	1	Yes	Yes
Q090	MRD-Pos	Ref	14	Dead	14	1	53	M	Yes	Adverse	1	Yes	No
Q091	MRD-Neg	CR	910	Alive	690	1	62	F	Yes	Adverse	0	No	Yes
Q092	MRD-Neg	CR	911	Alive	911	0	23	M	Yes	Adverse	0	Yes	Yes
Q093	MRD-Pos	CR	122	Dead	122	1	63	F	Yes	Intermed	1	Yes	No
Q094	MRD-Pos	Ref	1036	Alive	1036	0	70	F	Yes	Favorable	1	Yes	Yes
Q095	MRD-Pos	CR	108	Dead	108	2	42	M	Yes	Favorable	1	Yes	Yes
Q096	MRD-Pos	CR	383	Dead	327	1	76	F	Yes	Favorable	0	No	No
Q097	MRD-Pos	Ref	700	Alive	392	1	76	F	No	Intermed	0	No	No
Q098	MRD-Pos	CR	441	Dead	316	1	51	M	Yes	Adverse	1	Yes	Yes
Q099	MRD-Neg	CR	506	Alive	506	0	47	M	Yes	Adverse	1	Yes	No
Q100	MRD-Pos	Ref	516	Dead	224	1	56	M	No	Intermed	1	Yes	No
Q101	MRD-Pos	CR	797	Alive	797	0	73	F	Yes	Adverse	0	No	No
Q102	MRD-Neg	CR	449	Dead	415	1	43	M	Yes	Favorable	0	Yes	No
Q103	MRD-Pos	Ref	544	Dead	308	1	75	M	Yes	Intermed	1	No	No
Q104	MRD-Neg	Ref	885	Alive	885	0	31	M	Yes	Adverse	0	Yes	Yes
Q105	MRD-Pos	CR	667	Alive	667	0	58	F	Yes	Intermed	1	Yes	Yes
Q106	MRD-Pos	CR	1005	Alive	1005	0	46	F	Yes	Favorable	0	Yes	No
Q107	MRD-Pos	CR	918	Alive	266	1	63	M	No	Favorable	0	Yes	Yes
Q109	MRD-Neg	CR	861	Alive	861	0	41	M	Yes	Favorable	1	No	No
Q110	MRD-Neg	CR	1237	Alive	1237	0	47	F	Yes	Favorable	0	Yes	No
Q111	MRD-Neg	CR	146	Dead	146	2	63	F	Yes	Intermed	1	Yes	Yes
Q112	MRD-Neg	CR	251	Dead	161	1	53	F	Yes	Favorable	0	No	No
Q113	MRD-Pos	Ref	945	Alive	945	0	49	M	Yes	Adverse	0	Yes	Yes
Q114	MRD-Pos	CR	782	Alive	782	0	71	F	Yes	Adverse	0	No	Yes
Q115	MRD-Neg	CR	652	Alive	652	0	61	F	Yes	Favorable	0	Yes	No
Q116	MRD-Neg	CR	957	Alive	327	1	66	F	No	Favorable	0	Yes	Yes
Q117	MRD-Pos	Ref	42	Dead	42	1	70	F	No	Intermed	1	No	No
Q118	MRD-Neg	CR	644	Alive	257	1	39	M	Yes	Favorable	0	Yes	Yes
Q119	MRD-Neg	CR	902	Alive	361	1	35	M	Yes	Favorable	1	Yes	Yes
Q120	MRD-Pos	Ref	647	Alive	647	0	68	F	No	Adverse	0	Yes	Yes
Q121	MRD-Pos	Ref	140	Dead	140	1	70	F	No	Adverse	1	No	No
Q122	MRD-Neg	CR	1064	Alive	1064	0	58	M	Yes	Favorable	1	Yes	No
Q123	MRD-Pos	CR	74	Dead	74	2	61	M	No	Intermed	1	Yes	Yes
Q124	MRD-Pos	CR	223	Dead	222	1	50	F	Yes	Favorable	1	Yes	Yes
Q125	MRD-Pos	Ref	1110	Alive	1110	0	20	M	Yes	Adverse	1	Yes	Yes
Q126	MRD-Pos	CR	137	Dead	137	1	60	M	Yes	Adverse	0	Yes	No
Q127	MRD-Pos	Ref	105	Dead	105	1	74	F	No	Intermed	0	No	No
Q128	MRD-Neg	Ref	306	Dead	306	1	62	M	No	Adverse	0	Yes	No
Q129	MRD-Pos	CR	1228	Alive	1228	0	22	M	Yes	Favorable	0	Yes	No
Q130	MRD-Pos	CR	439	Dead	267	1	69	M	No	Adverse	0	Yes	Yes
Q131	MRD-Neg	CR	84	Dead	57	1	57	F	Yes	Adverse	1	Yes	No
Q132	MRD-Pos	Ref	931	Alive	931	0	63	F	Yes	Intermed	1	Yes	Yes
Q133	MRD-Neg	CR	545	Alive	545	0	60	M	Yes	Favorable	1	Yes	Yes
Q134	MRD-Neg	CR	533	Alive	366	1	57	M	Yes	Favorable	0	No	Yes

Table S3. Molecular MRD (by NGS) vs. treatment response by morphologic, cytogenetic, or flow cytometric analyses

	Number of Subjects (deaths)	
**Response to Induction Chemotherapy**	**MRD Negative (deaths)**	**MRD Positive (deaths)**
Complete Remission*	52 (18)	43 (25)
Refractory	7 (2)	26 (15)

* No detectable leukemia by traditional morphology, flow cytometry, and/or cytogenetics/ FISH
